# Albany Medical Annals’s Jubilee

**Published:** 1904-02

**Authors:** 


### Albany Medical Annals's Jubilee.

The *Albany Medical Annals* for January, 1904, is a jubilee number, consisting of 208 pages and contains 18 original articles besides editorial material, reviews, society proceedings, public health items, medical news and a synopsis of current literature, embracing the principal topics in medicine, including the specialties. The first communication relates to the *Medical Annals* and is an autobiographical sketch, prepared by Frederic C. Curtis and Willis G. Tucker. The second contribution, entitled Four cases of gangrene, is by Albert Vander Veer and Edgar A. Vander Veer, and is illustrated with four beautiful plates in colors. Other articles are Lipoma of the intestine, by Samuel B. Ward; Myasthenia gravis, by Henry Hun, George Blumer, and George L. Streeter; Acute ascending paralysis of the type of Landry, by Hermon C. Gordinier; A consideration of the efficacy of antitoxin in the treatment of diphtheria, by Joseph D. Craig; Nervous dyspepsia, by Andrew MacFarlane; The Islands of Langerhans in congenital syphilitic pancreatitis, by Richard Mills Pearce; A study of the changes occurring in the endometrium during the menstrual cycle, by H. Judson Lipes; The pathology and treatment of tetanus, with a report of three cases, by Arthur W. Elting; A plea for rational administration of chloroform as a routine measure in labor, by Spencer L. Dawes; the etiology and diagnosis of ozena and its relation to pulmonary tuberculosis, by Clement Theisen; Starch digestion in infancy, by Henry Larned Keith Shaw; The etiology of the summer diarrheas of children and of dysentery of bacterial origin, by Herbert D. Pease and Henry L. K. Shaw; Cysts of the mesentery, by Alvah H. Traver. The articles of Drs. Hun, Gordinier, Pearce, Lipes, Traver, and Root are illustrated.

The *Annals* begins its twenty-fifth year most auspiciously and publishes by far the most important single number of a medical magazine that we have yet seen, whether viewed from its scientific worth, literary excellence, or typographical beauty; and we desire to congratulate the editors as well as the publishers upon their enterprise and success.

---

THE advent of smallpox into a great city is always a menace. It becomes specially so in the present day when methods of intercommunication are so convenient and transit is so rapid. Travelers in large numbers pass to and fro on the great railway lines, many of whom are unprotected by vaccination and all of whom are liable to exposure from cases of walking smallpox. Trolley lines are especially dangerous as carriers of infection. These general observations are made at this time, because smallpox has made its appearance in Buffalo within the last few weeks, happily, however, only to a very limited extent. It is a preventable disease and the health department is amply competent to control the malady and stamp it out with promptitude, provided the necessary machinery is supplied by the common council. The health commissioner should be given all the inspectors and fumigators needed and the ordinances should be so constructed as to absolutely empower him with full authority to take prompt action in the face of menacing contagion. There is no economy in a parsimonious administration at such a time, and a certain autocratic power should be bestowed upon the department of health.

---

A NUMBER of congresses on tuberculosis have been announced in the medical journals. There is some danger that this subject will be overworked, unless the medical profession differentiates the proper scientific body to which it will give allegiance and sets to work to make the one selected representative in character. Dr.

S. A. Knopf, of New York, recently has sent out an open letter which has been published in the journals, discussing the subject in a calm and judicial manner. At a meeting scheduled to be held in Baltimore, January 25, 1904, under the auspices of the Maryland Tuberculosis Commission, it may be expected that some action will be taken, looking toward a settlement of the rival claims to recognition on the part of the several congress organisations. It appears reasonable to expect that the congress headed by Dr. Daniel Lewis, of New York, as president, scheduled to be held at Washington, April 4-6, 1905, is the one entitled to receive the support of the medical profession and of scientific workers throughout the country.

---

Last month we made editorial comment on the libel suit referred to in the following paragraph taken from the *Medical Times and Hospital Gazette* (London), December 19, 1903, which will prove of interest to all sympathisers of Dr. Bayliss.

Among other advantages to the cause of vivisection which have arisen out of the recent case of Bayliss vs. Coleridge is the fact that Messrs. Hempsons, the solicitors for Dr. W. M. Bayliss, have obtained from the publisher of a book called, The shambles of science, an apology for the libellous matters contained therein, and an undertaking that it shall be withdrawn from circulation and all copies handed over to the plaintiff's solicitors. We trust that this fact may obtain every possible publicity. The book in question is by Lizzy Lind Af. Hageby, and Leisa K. Schartau. The anti-vivisectionists just at present are having troublous times, and probably the last drop which makes their cup of bitterness to overflow is the news that a gentleman who desires to remain anonymous has, through Professor Starling, presented the sum of £50,000 to University College to be applied to the promotion of higher scientific education and research. Meanwhile, correspondence on the subject in certain daily papers is brisk, but up to the present no denial has appeared from Mr. Coleridge as to the rumor that someone else has paid his costs.

---

The optometry bill is again in evidence, this time in a most seductive manner. Elsewhere will be found an address to the profession by Dr. Frank Van Fleet, chairman of the committee on legislation of the Medical Society of the State of New York. The following will be read with interest, and should reach the eye of senators and assemblymen at Albany:

At a meeting of the Buffalo Academy of Medicine, held January 18, the following resolutions were unanimously adopted:

Whereas, Certain persons in this state have sent to physicians a copy of a proposed bill which will give to opticians official recognition by the board of regents, and other rights and privileges which belong to those who have completed the study of medicine as already prescribed by law, and

WHEREAS, In certain cases at least, very decided harm can be done to patients unless the person who prescribes glasses for them has a knowledge of medicine as well as optics, and

WHEREAS, The general proposition in the bill now proposed has already been condemned by the Medical Society of the State of New York, therefore

*Resolved,* That the Buffalo Academy of Medicine is unalterably opposed to giving the privilege of the practice of medicine to any persons not qualified for this responsibility by a knowledge of the fundamentals of medicine to-wit, anatomy, physiology, chemistry, pathology, physics and hygiene as demanded by the State Examining Boards, and

*Resolved,* That a copy of these resolutions be forwarded to our representatives at Albany and that they be requested to use all honorable means to defeat this measure.

